# Investigation on Law and Economics Based on Complex Network and Time Series Analysis

**DOI:** 10.1371/journal.pone.0127001

**Published:** 2015-06-15

**Authors:** Jian Yang, Zhao Qu, Hui Chang

**Affiliations:** 1 Department of Law, Tianjin University, Tianjin, China; 2 Department of English, Tianjin University, Tianjin, China; Tianjin University of Technology, CHINA

## Abstract

The research focuses on the cooperative relationship and the strategy tendency among three mutually interactive parties in financing: small enterprises, commercial banks and micro-credit companies. Complex network theory and time series analysis were applied to figure out the quantitative evidence. Moreover, this paper built up a fundamental model describing the particular interaction among them through evolutionary game. Combining the results of data analysis and current situation, it is justifiable to put forward reasonable legislative recommendations for regulations on lending activities among small enterprises, commercial banks and micro-credit companies. The approach in this research provides a framework for constructing mathematical models and applying econometrics and evolutionary game in the issue of corporation financing.

## Introduction

When small enterprises are raising capital for a project, they would take various factors into consideration. First of all, we assume that commercial banks and micro-credit companies provide almost the same amount of loan. Then the complexity of obtaining the loan and the application period will be the two most important factors. Correspondingly, various benefit factors and potential risks are the concerns of commercial banks and micro-credit companies, which will affect them to decide whether or not to offer loans to small enterprises. As a result, there exist a cooperative relationship and interactions in this social relation which is comprised of small enterprises, commercial banks and micro-credit companies. Furthermore, the relation network will directly influence each party’s strategy during the choosing process. This research is carried out to find out the relations between commercial banks and small enterprises, small enterprises and micro-credit companies so as to analyze the data generated from strategy choosing within the system and offer legislative recommendations for lending activities. According to relevant literature, research on fund raising are mostly conducted from the perspective of one or two parties of small enterprises, commercial banks and micro-credit companies [1–3]. Game theory, especially evolutionary game have been used to simulate the interactions and explore the cooperative behaviors among players on the square lattice [4–6]. In this paper, evolutionary game was used to integrate the three parties and model the interactions among them. Through the analysis of time series, the relation network within the social system can be revealed. On that basis, qualitative analysis of the relation network was made and the legal norms for lending activities were conducted, which is rare and innovative in the field of corporation financing research.

During the past few years, the complex network theory has gone through remarkable progress and the study of complex network has become an interdisciplinary subject which arouses extensive attention from various disciplines. In a complex network, the components are considered as nodes and edges representing the interactions among nodes. Therefore, the complex network is the mathematical representation of complex systems. Many advanced complex network approaches have been proposed to analyze univariate time series [7–11] and multivariate time series [12–14]. Complex network analysis has been successfully applied in many research fields, such as network topology estimation [15–17], financial system [18], climate system [19], gene system[20], grain property networks [21–22], multiphase flow system [23–25], and friction networks in nucleation processes [26–27], all of those have demonstrated their strength in characterizing real complex systems from time series. Moreover, complex network has been applied in spreading dynamics [28–29] and topology identification [30–32].

In this research, a real social network was set up and compressive sensing was used to reconstruct the complex network through evolutionary game data. Based on time series of certain observable quantities generated from observations and experiments, revealing network topology is very important. It is the first time that complex network has been used in law and economics to reveal the relation network.

## Methods and Technologies

According to the method proposed by Professor Wang Wenxu etal.[17], the hidden network can be revealed. In order to uncover network topology existing among small enterprises, commercial banks and micro-credit companies, this method is used as reference. The core concepts are as follows. This exemplification for sparse-signal reconstruction is recently developed and has been applied in a broad range. Compressive sensing is one type of convex optimization and it has been used to reconstruct coupled-oscillator networks. This method emphasize the advantages of compressive sensing, for example, when addressing the inverse problem of network reconstruction which is based on continuous or discrete data, it needs only a small amount of data. We transform the problem of uncovering the network typology into that of sparse-signal reconstruction by presenting a mathematical framework. In a typical game, in order to gain maximum payoff, agents choose different strategies and usually, the strategies can be divided into two types: cooperation and defection. By using this method which based on compressive-sensing, we can generate exact knowledge of the node-to-node interaction pattern in high efficiency, even when the number of the available data of each agent’s strategy and payoff is limited. We implement our method through (1) extensive numerical computations by using model complex networks and evolutionary games, and (2) a real social experiment where agents form a network by playing a typical game and produce short sequence data of strategy and payoff. This method is appropriate for revealing “hidden” networks existed in different social, economic and biological systems owing to the high prediction accuracy and the distinct extremely small data requirement.

In an evolutionary game, a player usually can choose one of two strategies(S), at any time: cooperation(C) or defection (D). It can be expressed as S(C) = (1,0)^T^ and S(D) = (0,1)^T^_,_ in which T represents “transpose”. The payoffs of two related players are determined by their strategies and payoff matrix. For instance, for the prisoner’s-dilemma game (PDG) and the snowdrift game (SG), the payoff matrices are

$${\text{P}}_{\text{PDG}}=\left(\begin{array}{cc} \text{1} & \text{0}\\ \text{b} & \text{0} \end{array}\right)\text{ or }{\text{P}}_{\text{SG}}=(\begin{array}{cc} 1 & 1-\text{r}\\ \text{1}+\text{r} & \text{0} \end{array}),$$ (1)

in which b(1<b<2) and r(0<r<1) are parameters characterizing the temptation to defect. When a player who chooses defection encounters a player who chooses cooperation, the defector’s payoff is b in the PDG, and 1+r in the SG, while the payoff of the cooperator is 0 in the PDG and 1-r in the SG. In every step, all agents play the game with their neighbors and obtain payoffs. For agent i, the payoff is

$$G_{i}\;=\;\underset{j\in \Gamma_{i}\;}{\Sigma }S_{i}^{T}PS_{j},$$ (2)

in which S_i_ and S_j_ stands for strategies of agent i and j at that point, and the sum is over the neighbor-connection set Γ_i_ of i. After gaining the payoff, in order to maximize the payoff of next round, each agent renovates its strategy, which is in accordance with its own and its neighbors’ payoffs. To acquire an agent’s decision-making process, there are applicable mathematical rules, such as the best-take-over rule, the Fermi equation, and payoff-difference-determined updating probability. Specifically, in our simulations of evolutionary-game dynamics and yield time series, we employ the Fermi rule accordingly. We define that after an agent i choose a neighbor j randomly, the possibility that i adopts j’s status S_j_ is

$$W(S_{i}\leftarrow S_{j})=\frac{1}{1+\text{exp}[(G_{i}-G_{j})/\kappa ]’}$$ (3)

in which κ represents the stochastic uncertainties in the game dynamics. For instance, if G_j_<G_i_ κ = 0 equals to complete rationality where the possibility is 0, and the possibility is1 if G_i_<G_j_, and κ→∞ equals to absolute random decision making. Therefore, the possibility W represents the player’s delimited rationality in society and the natural decision making which is relatively fitness-based in evolution.

The purpose of compressive sensing is to reconstruct a X ∈ R^N^ from liner measurements Y about X in the form Y = Φ · X where Y∈R^m^ and Φ is an M ×N matrix. The prominent characteristic is that the number of measurements is much lesser than the number of components of the unknown vector, i.e., M<<N. By solving the following convex-optimization problem: min ǁ X ǁ_1_ subject to Y = Φ · X, in which ${\left\|X\right\|}_{1}=\sum {}_{i=1}^{N}\left|X_{i}\right|$ is the L_1_ norm of vector X, we can make accurate reconstruction and there are available solutions to the convex-optimization problem. In this paper, we would prove that compressive sensing method can solve network-construction problems different from oscillator networks by using the small amount of data from evolutionary games.

When solving the compressive sensing, the most critical part is the connection between players’ payoffs and strategies. We can characterize the interactions among players in the network by an N ×N adjacency matrix A with elements a_ij_ = 1 if there is a connection between players i and j, otherwise, a_ij_ = 0. The payoff of agent x can be conveyed by:

$$\begin{array}{l} G_{\text{x}}\left(\text{t}\right)={\text{a}}_{\text{x1}}S_{\text{x}}^{T}\left(\text{t}\right)\text{ · }\underset{}{P_{}}\text{·}S_{\text{1}}\left(\text{t}\right)+...+{\text{a}}_{\text{x, x-1}}S_{\text{x}}^{T}\left(\text{t}\right)\text{ · }\underset{}{P_{}}\text{·}S_{\text{x-1}}\left(\text{t}\right)\\ +{\text{a}}_{\text{x, x}+\text{1}}S_{\text{x}}^{T}\left(\text{t}\right)\text{ · }\underset{}{P_{}}\text{·}S_{\text{x}+\text{1}}\left(\text{t}\right)+...+{\text{a}}_{\text{xN}}S_{\text{x}}^{T}\left(\text{t}\right)\text{ · }\underset{}{P_{}}\text{·}S_{\text{N}}\left(\text{t}\right), \end{array}$$ (4)

in which a_xi_(i = 1, · · ·, x-1, x+1, · · ·, N) stands for a possible relation between agent x and its neighbor i;

$$a_{xi}S_{x}^{T}\left(t\right)\cdot P\cdot S_{i}\left(t\right)\left(i=1,...,x-1,x+1,...,N\right)$$ (5)

represents agent x’s possible payoff from the evolutionary game with i (if there is no relation between x and i, a_xi_ = 0 and thus the payoff is zero); and t = 1, · · ·, m is the number of the rounds that all agents play with their neighbors. The relation shows the base to construct the vector G_x_ and matrix Φ_x_ in a suitable compressive sensing framework to acquire a solution of player x’s neighbor-connection vector A_x_. Before all, we write

$$\Phi_{\text{x}}=\left[\begin{array}{cccccc} F_{x1}\left(t_{1}\right) & ... & F_{x,x-1}\left(t_{1}\right) & F_{x,x+1}\left(t_{1}\right) & ... & F_{xN}\left(t_{1}\right)\\ F_{x1}\left(t_{2}\right) & ... & F_{x,x-1}\left(t_{2}\right) & F_{x,x+1}\left(t_{1}\right) & ... & F_{xN}\left(t_{2}\right)\\ ... & ... & ... & ... & ... & ...\\ F_{x1}\left(t_{m}\right) & ... & F_{x,x-1}\left(t_{m}\right) & F_{x,x+1}\left(t_{1}\right) & ... & F_{xN}\left(t_{m}\right) \end{array}\right]$$ (6)

$$G_{\text{x}}={\left(G_{x}\left(t_{1}\right),G_{x}\left(t_{2}\right),\cdot \cdot \cdot ,G_{x}\left(t_{m}\right)\right)}^{T}\;$$ (7)

and

$$A_{\text{x}}={\left({\text{a}}_{\text{x1}},\;\cdot \cdot \cdot \;{\text{a}}_{\text{x, x-1}},\;{\text{a}}_{\text{x, x}+\text{1}},\;\cdot \cdot \cdot ,\;{\text{a}}_{\text{xN}}\right)}^{T}$$ (8)

in which $F_{xy}\left(t_{i}\right)=S_{x}^{T}\left(t_{i}\right)\cdot P\cdot S_{y}\left(t_{i}\right)$. The vectors G_x_, A_x_ and matrix Φ_x_ conform to G_x_ = Φ_x_ · A_x_. The sparsity of A_x_ makes the compressive-sensing framework available. The vector G_x_ can be acquired from the payoff data directly. The matrix Φ_x_ can be generated from the strategy because $S_{x}^{T}\left(t_{i}\right)$ and *S*_*y*_(*t*_*i*_) in *F*_*xy*_(*t*_*i*_) come from data and P is known. As a result, the vector A_x_ can be predicted through time series alone. Similarly, we can predict all agents’ neighbor-connection vectors, generating the network adjacency matrix A = (A_1_, A_2_, ···, A_N_).

We use model complex networks to testify our approach by employing PDG on three types of complex networks: random, small-world, and scale-free. During the system’s evolution towards the steady state, we record time series of strategies and payoffs to reveal the topology of the interaction network. We implement the success rates of existent links (SREL) and nonexistent links (SRNL) to quantify the performance of the amount of required measurements. If the predicted value of an element of the adjacency matrix A approaches to zero, we regard there is no connection, otherwise, there exists corresponding connection if the value approaches 1. In reality, we arrange the threshold smaller, e.g., 0.1, then the range of existent links is 1 ± 0.1 and the range of nonexistent links is 0 ± 0.1. Any value beyond the two sections is supposed as a failure of the prediction. For a single player, SERL is defined as the ratio of successfully predicted neighbor-connection links to that of actual neighbors and SRNL is defined in a similar way. Then we acquire the values of SREL and SRNL by averaging all nodes for the whole network. Due to the sparsity of the potential complex network where the number of nonexistent links is usually larger than that of existent links, we treat SREL and SRNL separately. Under the circumstance that the chosen threshold is neither too close to 1, nor to zero, it slightly affects the overall success rates.

## The application in a social complex network

### Ethics statement

The experiment was approved by Tianjin University. The approval number is 2013XRG-0106. All participants provided their written informed consent.

In this research, the above method was used to reveal a real social complex network. The network was set up among small enterprises, commercial banks and micro-credit companies. In the experiment, 22 participants from Tianjin University represented complex network nodes, and they interactively played PDG together. Participants No.0 to No.5 represented commercial banks; participants No.10 to No.19 represented small enterprises and participants No.20 to No.25 represented micro-credit companies. Among all the participants, if a small enterprise had already been acquainted with a bank or a micro-credit company prior to the experiment, there was a social tie (link) between them; otherwise, there was no link. During the experiment, all participants could change the strategies in each round to maximize the payoff, but they were not allowed to communicate with other agents, so the changing of strategies was based on the agent’s own inference. The strategies and corresponding payoffs for each agent are shown in Tables 1 and 2. When completing one round of games, each agent recorded his or her payoff and then chose the strategy for next round, either continuing the strategy of former rounds or changing that into another one. Finally, all participants completed 40 rounds of games and their strategies and payoff of each round served as the available database for prediction and the data used for reconstruction was selected randomly.

**Table 1:** The strategy and corresponding payoff between small enterprises and commercial banks.

Small enterprises (X)		Commercial banks (Y1)	
Strategy	Payoff	Strategy	Payoff
A	1	H	1
B	1.5	H	-1
A	-0.5	G	0.2
B	-0.5	G	0.2
C	0.2	G	0
C	0.2	H	0

X: small enterprises; Y1: commercial banks

**Table 2:** The strategy and corresponding payoff between small enterprises and micro-credit companies.

Small enterprises (X)		Micro-credit companies (Y2)	
Strategy	Payoff	Strategy	Payoff
A	0.8	H	1.5
B	1.2	H	-0.7
A	-0.5	G	0.2
B	-0.5	G	0.2
C	0.2	G	0
C	0.2	H	0

X: small enterprises; Y2: micro-credit companies

## Results and Analysis

### 4.1 The Experimental Results

**Fig 1:**
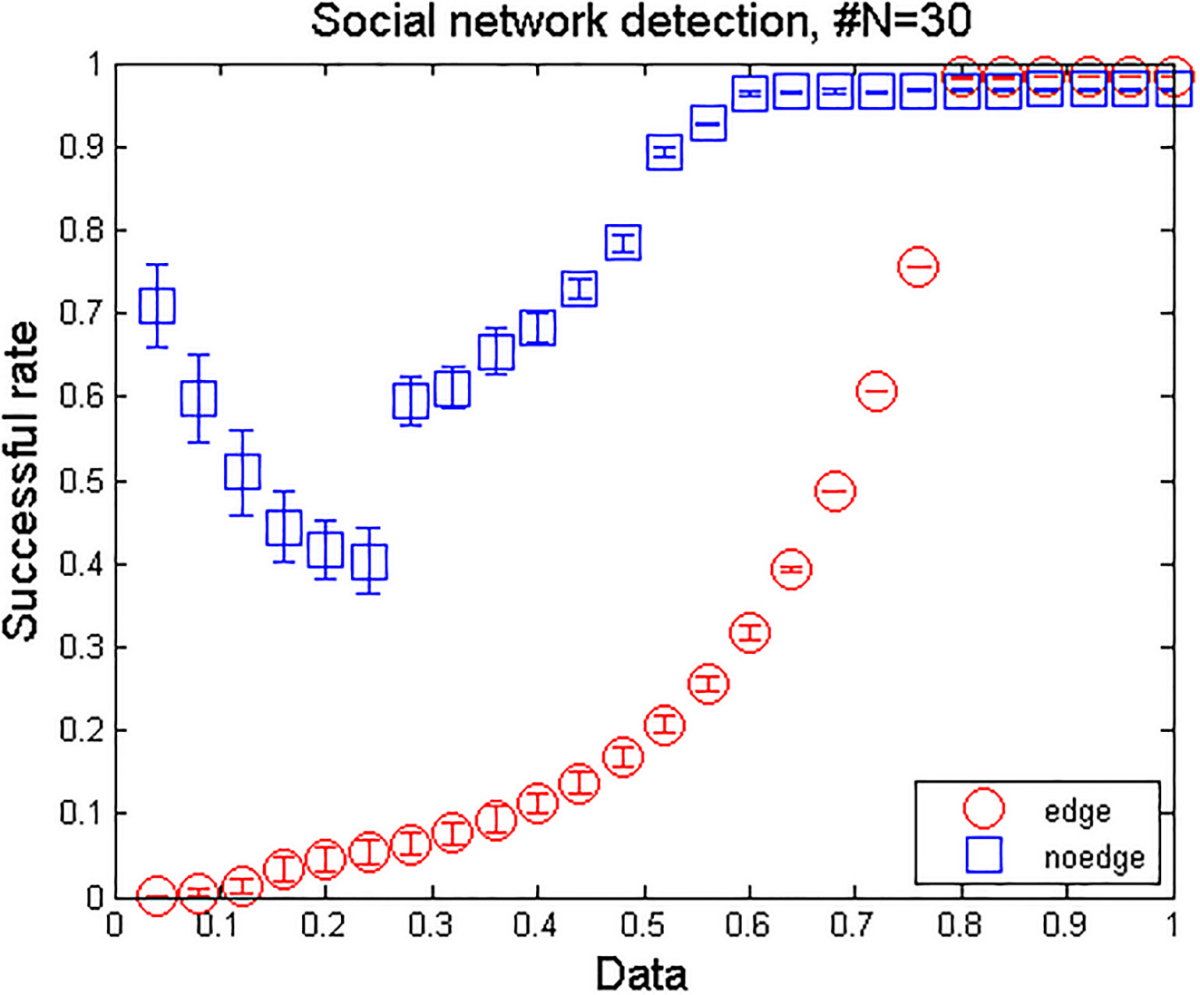
Success rate of revealing social network topology. The network size is 22. For each realization, measurements are randomly picked from a time series of temporary evolution. The error bars denote the standard deviations.

**Fig 2:**
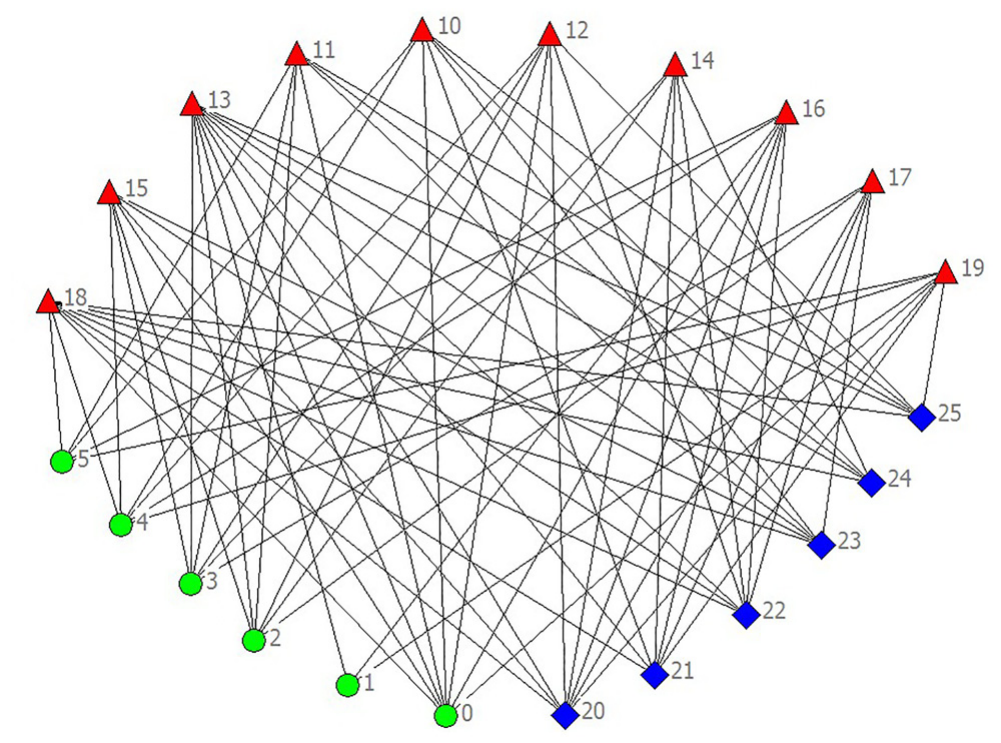
The network structure among all participants, in which triangles represent small enterprises, circles represent commercial banks and diamonds represent micro-credit companies.

The result is shown in Fig 1. Although the decision-making process of each participant was complicated, the uncovering of social network is successful. The length of data which is used to achieve 100% success rate in the prediction of social ties is about 0.8, which is larger than the simulation results. The relative smaller size and denser connections in real social networks than in model networks are the probable causes. The revealed network structure is illustrated in Fig 2.

### 4.2 Analysis from the Perspective of Small Enterprises

For small enterprises, different strategies may lead to the following different results.

1. Applying for loans and pay loans on schedule. The payoff shows a degree of fluctuation and the frequency of getting high income is relatively low.
2. Applying for loans but default on loans. Small enterprises have relatively more chances to get high income but the payoff exhibits the greatest fluctuation, compared with other strategies.
3. Choosing not to apply for loans and operate projects relying solely on their own fund. The fluctuation is slight but at the same time, the possibility of getting high income is low.

**Fig 3:**
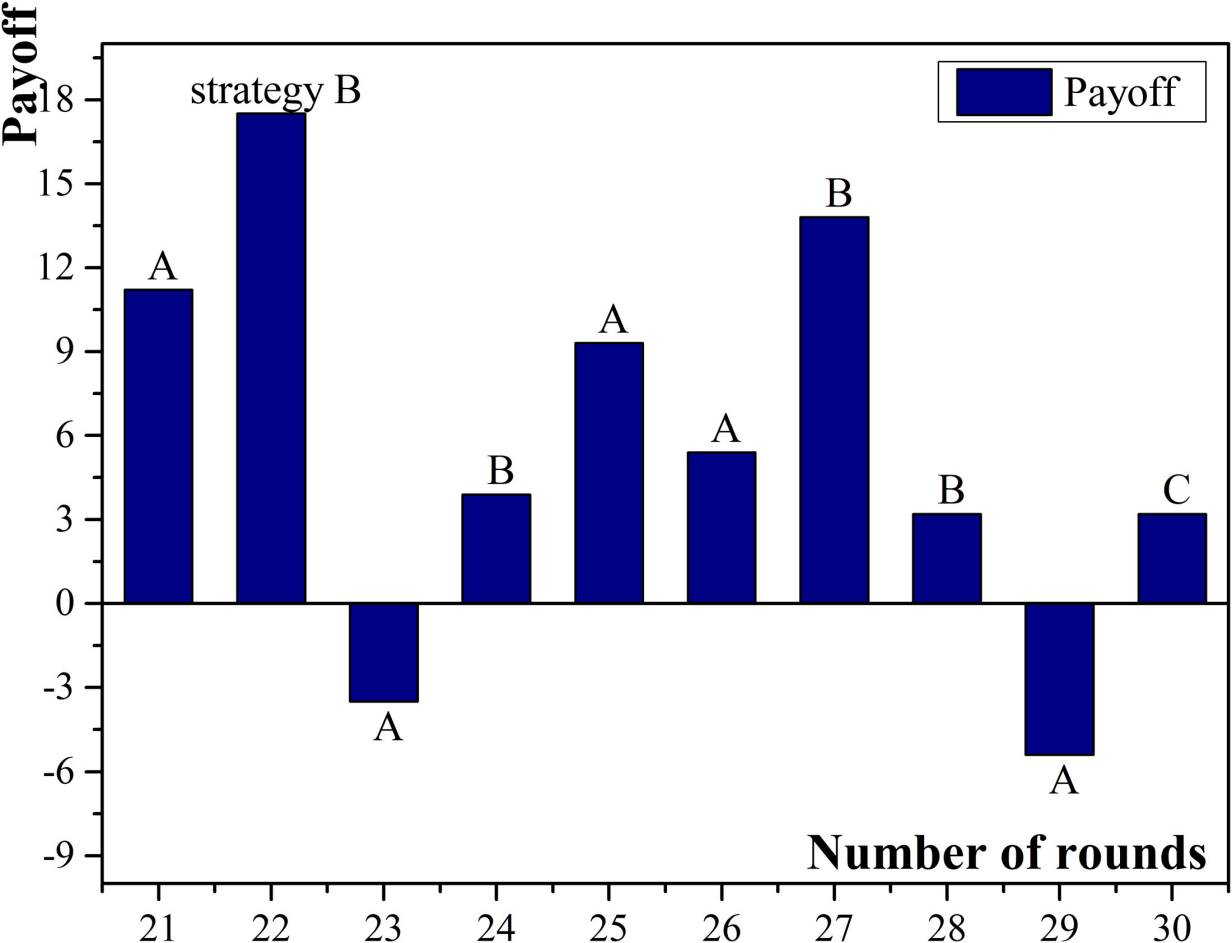
Payoffs of small enterprise No.18.

**Fig 4:**
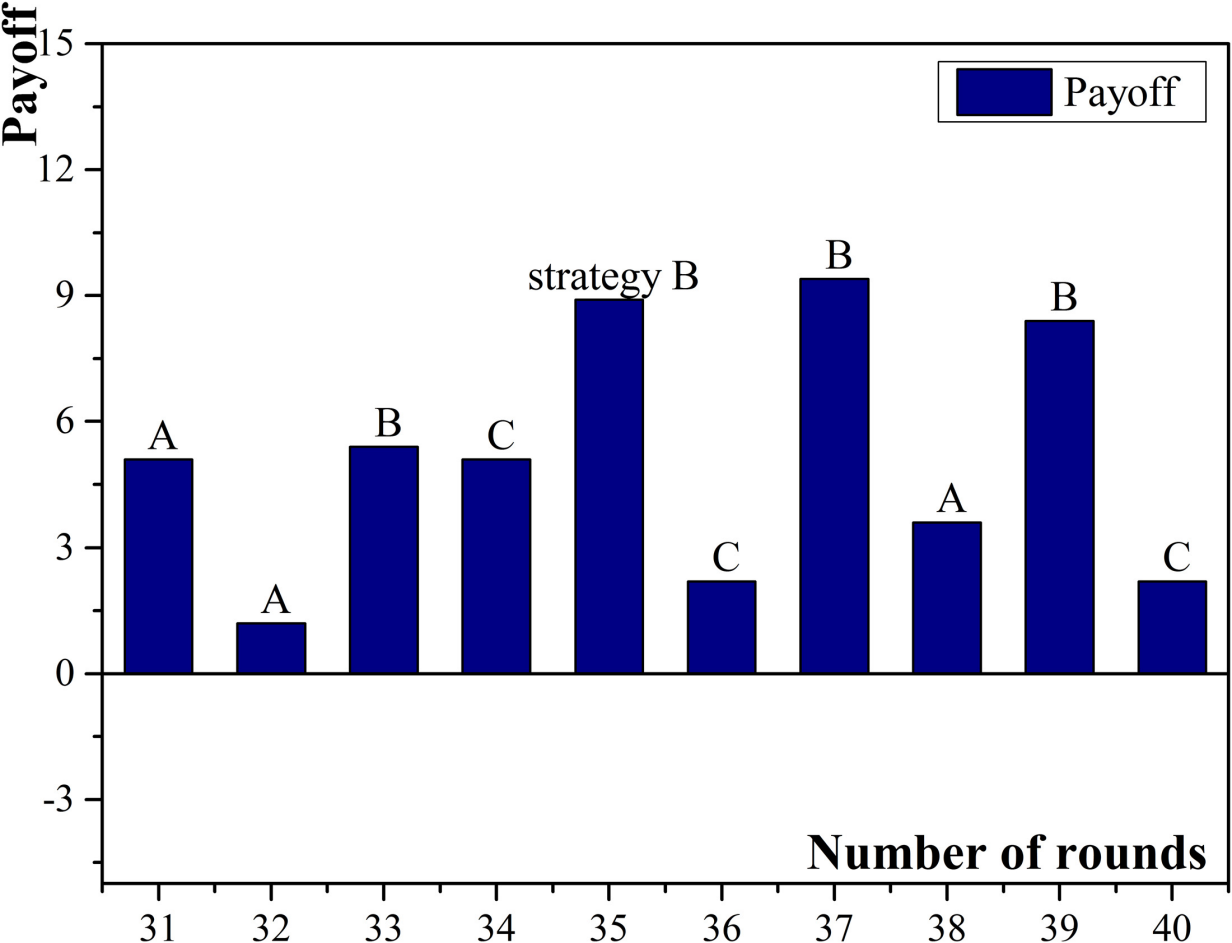
Payoffs of small enterprise No.16.

The experimental results show that the more neighbors a small enterprise has, the higher possibility of acquiring high income it has, especially when micro-credit companies are chosen as neighbors. For example, agent No.18 had 9 neighbors and the number was the largest among all participating small enterprises. Besides, all the micro-credit companies were chosen as the neighbors and the normalized payoff of agent No.18 was the highest. The probable reasons are: (1) Small enterprises have relatively higher chances getting high income when they apply for but default on loans; (2) Compared with commercial banks, micro-credit companies have more possibility and preference to provide loans. Therefore, small enterprises which have more connected micro-credit companies will have more chances to obtain loans and then, if they choose to default on loans, the income may become high. Agent No.18 obtained the payoff higher than 10 for seven rounds and there were six times when it chose to apply for but default on loans. Compared with agent No.18, agent No.16 had more connected commercial banks than connected micro-credit companies in the neighbors and the amount of connected micro-credit companies was three, which was the least among all small enterprises. The normalized payoff of agent No.16 was the lowest because commercial banks had less frequency than micro-credit companies to choose cooperation, and thus it was more difficult to obtain sufficient loans. The payoff diagram of agent No.18 and No.16 are shown in Fig 3 and Fig 4.

Loan cost, application period and the possibility of acquiring loans are small enterprises’ major concerns. On account of the low loan interest rate, commercial banks may become small enterprises’ first choice for financing. While in consideration of commercial banks’ cautious attitude towards petty loan and complicated procedure of loan application, small enterprises may turn to micro-credit companies. Despite the higher loan interest rate, micro-credit companies possess advantages of higher possibility of loan approval, flexibility and shorter application period, which make it more convenient for small enterprises to achieve capital turnover quickly. As a result, even the related commercial banks refuse to provide loans, small enterprises may still apply for loans which is to win the cooperation of micro-credit companies. Then the income brought by the cooperation can compensate to the potential loss caused by the defection of related commercial banks. Small enterprises want to get the highest income under the acceptable risk level, therefore after obtaining loans, they may default on loans which may bring higher income as well as the risk of the falling of credit rating, which will cause neighbors to refuse to give credit in the future. When the payoff exhibits large fluctuation or the frequency of getting high income is less, small enterprises would choose to operate the project relying on their own fund, rather than financing.

Based on the experimental results and current situation, there will be two possible conditions in which small enterprises choose to apply for loans but breach contracts. Firstly, delaying loan payment, or taking the measure called “repaying for borrowing” to alleviate the financial stress. Small enterprises shall be liable for breaching of contracts and paying liquidated damages to commercial banks. Meanwhile, these enterprises in default need assume the falling of credit level caused by their loan defaults and the potential loss due to the difficulty of acquiring loans in future operation. Another situation is that small enterprises default on loans because of the bankruptcy. In accordance with *Company Law of the People's Republic of China(2013)*[33], *Regulations of the People's Republic of China on Administration of Company Registration (Revised 2014)*[34], *Regulations of the People's Republic of China for Controlling the Registration of Enterprises as Legal Persons (Revised 2014)*[35], there is almost no clear stipulation concerning whether the shareholders, executive officers or legal person of the newly established enterprises had ever managed enterprises and the credit situation of those enterprises. According to *Law of the People's Republic of China on Enterprise Bankruptcy (2006)* [36], the insolvent assets shall, after the costs for bankruptcy liquidation and community liabilities are repaid in priority. According to the discharge sequence, the common credits will be the last item to be paid. Under such circumstances, there exists the possibility that small enterprises exploit the loopholes of laws, which is, filing for bankruptcy to avoid credit repayment and then establish a new enterprise which would not be influenced by previous debts. Small enterprises shall also assume the breach cost but the cost is relatively lower while commercial banks and micro-credit companies will face greater loss. In response, it is highly recommended that relevant laws and regulations make clear stipulation so that the profit of financial institutions which provide loans can be guaranteed.

### 4.3 Analysis from the Perspective of Commercial Banks

From the perspective of commercial banks, disparate strategies may lead to the following different results.

1. Choosing cooperation. The payoff shows large fluctuation. The highest income is slightly lower than that of micro-credit companies and the frequency of loss is higher than that of micro-credit companies. When the loss appears, commercial banks would immediately change the strategy into defection in order to guarantee the basic benefit.
2. Choosing defection which means refusing to provide loans. The income is relatively low and the fluctuation is small. When continuously obtaining the low income, commercial banks may choose cooperation in order to increase the income, but when the great loss appears, they will immediately change the strategy of next round into defection.

**Fig 5:**
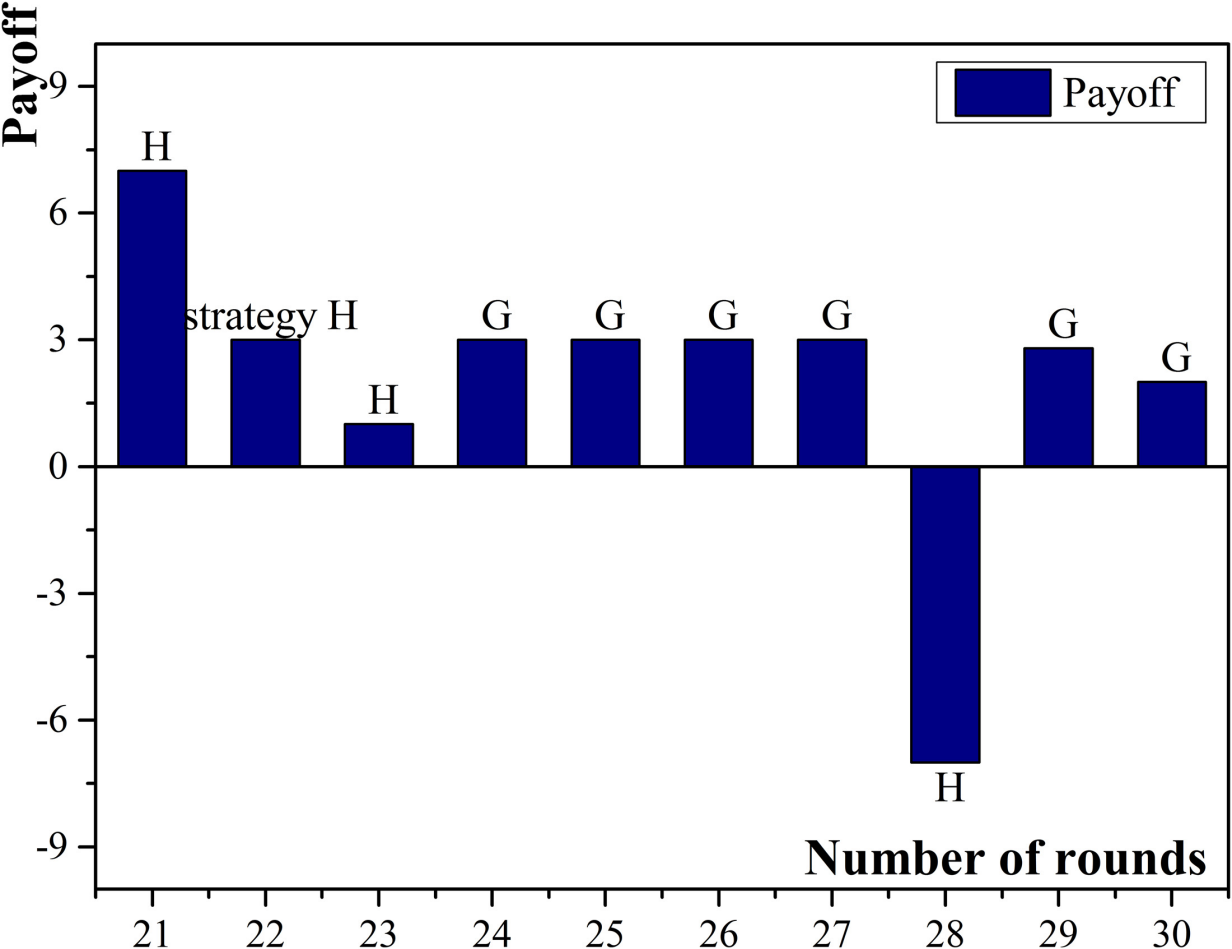
Payoffs of commercial bank No.0.

**Fig 6:**
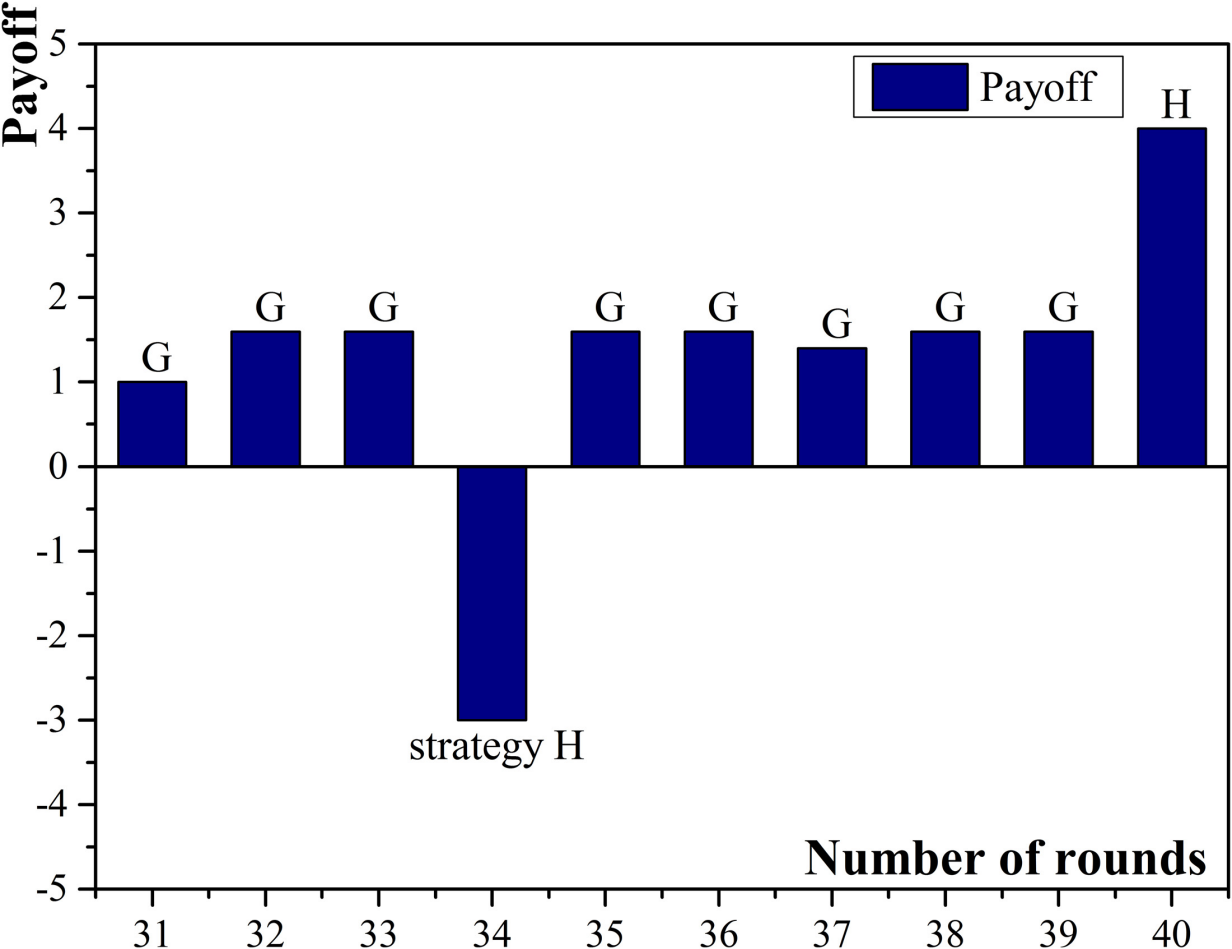
Payoffs of commercial bank No.1.

During the experiment, agent No.0 had 8 connected small enterprises which was the highest in all commercial banks and the normalized payoff was the largest. There were 11 rounds in all that agent No.0 chose approving the loan and 7 times that it got positive return. Agent No.0 continuously chose cooperation for 4 rounds where two of the connected small enterprises chose applying for loans and paying loans on schedule. Because of the above two “good credit” enterprises, agent No.0 kept obtaining positive return. With the number of “good credit” borrowers increasing, agent No.0 got higher income. When the above two small enterprises did not pay loans on schedule at the same time, which may reduce the payoff of agent No.0, so it would change to defection in later rounds. Agent No.1 had the smallest number of related small enterprises, with only three. As a result, agent No.1 easily suffered from the loss caused by related small enterprises’ loan defaults. Even when choosing defection, the possibility of acquiring high income may still be low so the normalized payoff of agent No.1 was the lowest among all the participated commercial banks. The payoff diagram of agent No.0 and No.1 are shown in Fig 5 and Fig 6.

Loan profit and loan safety are commercial banks’ major focus. Commercial banks chose cooperation so that they could make good use of the capital and get high benefit. When confronting the situation that individual small enterprises choose defaulting on loans, commercial banks will consider other related small enterprises’ credit level and the possible income of cooperation. Commercial banks may choose defection when facing loan defaults from small enterprises or the number of cooperative small enterprises with high credit level was small or some of those enterprises chose defaulting,. If the positive return can compensate the loss led by small enterprises’ loan defaults, commercial banks may still choose cooperation. Through taking the measures like strict examination and verification of loan qualifications and repayment supervision, commercial banks’ benefit can be guaranteed. In summary, commercial banks would make good use of capital and will not continuously or frequently choose defection because the opportunity loss caused by the idle fund should be avoided.

### 4.4 Analysis from the Perspective of Micro-credit Companies

From micro-credit companies’ view, various strategies may lead to the following different results.

1. Choosing cooperation which means providing loans. The payoff exhibits large fluctuation. The highest income is higher than that of commercial banks and the frequency of loss is relatively less than commercial banks.
2. Choosing defection which means refusing to provide loans. The income is relatively low and the fluctuation is small. In order to obtain high income and win over more clients, micro-credit companies prefer cooperation to defection.

**Fig 7:**
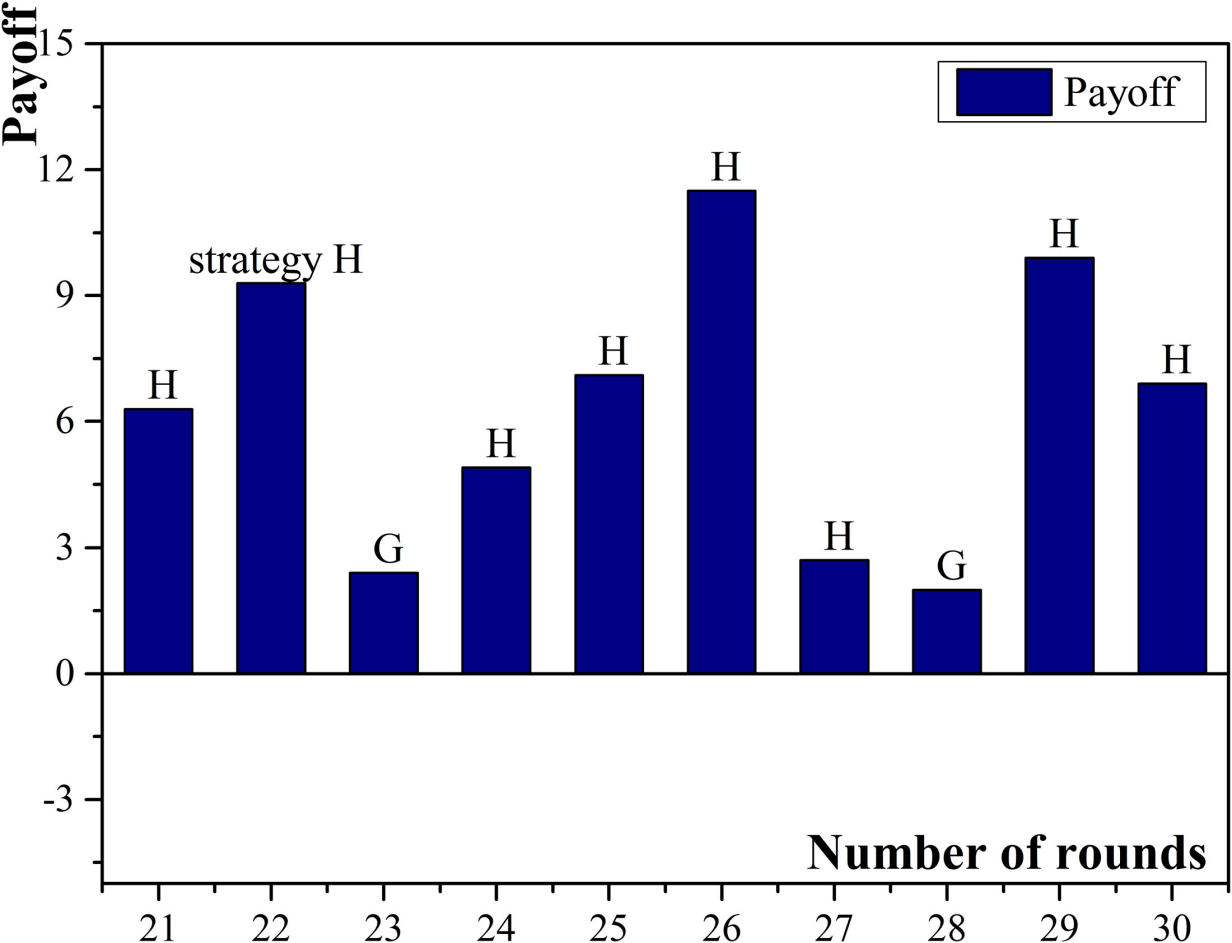
Payoffs of micro-credit company No. 20.

**Fig 8:**
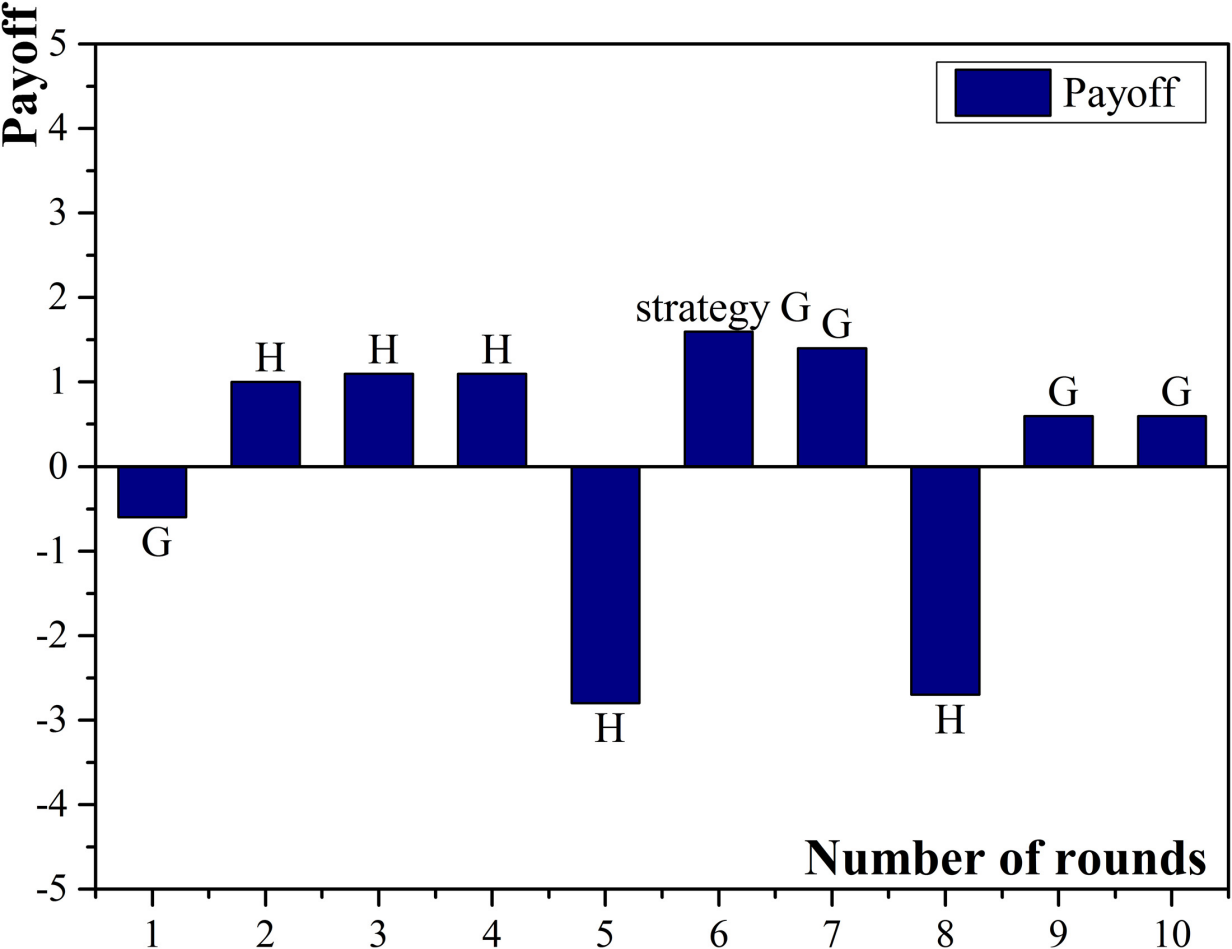
Payoffs of micro-credit company No. 25.

Petty loan is micro-credit companies’ main business, so compared with commercial banks, micro-credit companies have more frequency in choosing cooperation among all micro-credit companies. Agent No.20 had the largest amount of neighbors, with 8 related small enterprises. The strategy of cooperation was chosen for 25 times and the normalized income was the highest among all participated micro-credit companies. There were 3 related small enterprises choose applying for loans and paying loans on schedule in high frequency, so the basic income of agent No.20 can be guaranteed. Under the above situation, micro-credit companies preferred sustainable cooperation in order to win over the cooperation from other clients. Agent No.25 had six neighbors which was the smallest. Compared with other micro-credit companies, it had less neighbors who chose applying for loans and paying loans on schedule. Combining with its less frequency of choosing cooperation, the normalized payoff of agent No.25 was the lowest. The payoff diagram of agent No.20 and No.25 are shown in Fig 7 and Fig 8.

The factors that micro-credit companies focus on are similar with that of commercial banks. When confronting the situation that individual small enterprises choose defaulting on loans, micro-credit companies will consider other related small companies’ credit level and the possible income of cooperation. If the positive return can compensate the loss caused by neighbors’ loan defaults, micro-credit companies may still approve the application for loan. Compared with commercial banks, micro-credit companies expect more about the capital turnover in high speed and they prefer to provide loans so that they can win over more cooperation from other enterprises. Furthermore, due to the short application period, if the cooperated small enterprises have financial problems, the guarantee’s depreciated surplus value that micro-credit companies can acquire is relatively higher. Furthermore, flexible measures for recourse can be immediately taken to guarantee the basic profit.

According to People’s Bank of China and banking supervision and management industry’s regulations on small loan company’s registered capital and fund application, combing with People’s Bank of China’s regulations on petty loans’ limit, the amount of loan that micro-credit companies and commercial banks can provide is almost the same. The loan interest rate is also be regulated accordingly. The source of capital differs. For micro-credit companies, the source and the amount of capital are limited, while for commercial banks, they have more capital sources and the capital is better guaranteed. Owing to the above difference, the management model of micro-credit companies and commercial banks are various. In order to increase the number of clients, micro-credit companies take advantages of flexible loan pattern and shorter loan approval period which satisfy small enterprises’ needs of quickly achieving capital turnover. Meanwhile, micro-credit companies’ flexible measures for calling in loans are used to guarantee its basic profit. As traditional financial institutions, commercial banks have sufficient capital and the loan interest rate is relatively low. The operation and supervision on loan transaction are more normative and efficient. Commercial banks are cautious about the allocation of capital because petty loan is only a part of the whole loan transaction. In terms of recovering loans, more systematic and normative measures are taken to guarantee their basic income. Thus, measures like involving micro-credit companies into central bank’s credit reporting system and raising micro-credit companies’ financing scale and scope to promote the fair competition between micro-credit companies and commercial banks are greatly recommended.

## Conclusion

In this paper, evolutionary game has been used to involve small enterprises, commercial banks and micro-credit companies together, stimulating the decision-making process in financing activities. Based on compressive sensing, a method has been proposed to reveal the complex relation network within the social system. Through analyzing time series generated from the experiment, our method has successfully achieved the reconstruction by using only a small amount of data. The results perfectly match the current situation that small enterprises have default tendency in lending activities. Accordingly, commercial banks and micro-credit companies have their own considerations about loan costs, potential risks and benefits of approving petty loans. Therefore each party will choose reasonable strategies to maximize its own return. According to the results and taking the current laws and regulations as reference, appropriate recommendations for legal norm aiming at the lending activities among small enterprises, commercial banks and micro-credit companies have been proposed.
